# Genotypic spectrum underlying tetrahydrobiopterin metabolism defects: Experience in a single Mexican reference center

**DOI:** 10.3389/fgene.2022.993612

**Published:** 2022-10-12

**Authors:** M. Vela-Amieva, M. A. Alcántara-Ortigoza, I. Ibarra-González, A. González-del Angel, L. Fernández-Hernández, S. Guillén-López, L. López-Mejía, R. I. Carrillo-Nieto, M. O. Fiesco-Roa, C. Fernández-Lainez

**Affiliations:** ^1^ Laboratorio de Errores Innatos del Metabolismo y Tamiz, Instituto Nacional de Pediatría, Secretaría de Salud, Ciudad de México, México; ^2^ Laboratorio de Biología Molecular, Instituto Nacional de Pediatría, Secretaría de Salud, Ciudad de México, México; ^3^ Unidad de Genética de la Nutrición, Instituto de Investigaciones Biomédicas, UNAM, Ciudad de México, México; ^4^ Laboratorio de Citogenética, Instituto Nacional de Pediatría, Secretaría de Salud, Ciudad de México, México; ^5^ Maestría y Doctorado en Ciencias Médicas y de la Salud, UNAM, Ciudad de México, México

**Keywords:** hyperphenylalaninemia, phenylketonuria, 6-pyruvoyl-tetrahydropterin synthase, dihydropteridine reductase, pterin-4-alpha-carbinolamine dehydratase, inborn errors of metabolism, rare diseases

## Abstract

**Background:** Pterin profiles or molecular analyses of hyperphenylalaninemia (HPA) caused by phenylalanine hydroxylase (PAH) deficiency or tetrahydrobiopterin deficiency (BH4D) are not always available in low- or middle-income countries, including Mexico, limiting information regarding the phenotypic and genotypic characteristics of patients exhibiting BH4D.

**Objective:** To report the genotypes underlying BH4D and the clinical presentation in unrelated Mexican HPA pediatric patients with normal *PAH* genotypes who attended a single metabolic reference center in Mexico.

**Methods:** Automated Sanger sequencing of the *PTS*, *QDPR*, and *PCBD1* genes of 14 HPA patients was performed. Predicted effects on protein structure caused by missense variants were assessed by *in silico* protein modeling.

**Results and discussion:** A high prevalence of BH4D was noted in our HPA cohort (9.8%, N = 14/142). Clinically relevant biallelic genotypes were identified in the *PTS* (N = 7/14 patients), *QDPR* (N = 6/14 patients), and *PCBD1* (N = 1/14 patients) genes. Four novel *QDPR* variants [c.714dup or p.(Leu239Thrfs*44), c.106-1G>T or p.(?), c.214G>T or p.(Gly72*), and c.187_189dup or p.(Gln63dup)] were identified. *In silico* protein modeling of six missense variants of *PTS* [p.(Thr67Met), p.(Glu81Ala), and p.(Tyr113Cys)], *QDPR* [p.(Cys161Phe) and p.(Pro172Leu)], and *PCBD1* [p.(Glu97Lys)] supports their pathogenicity. Progressive neurological symptoms (mainly intellectual and motor impairment and even death in three patients) were noted in all patients with biallelic *QDPR* genotypes and in 5/7 patients bearing biallelic *PTS* genotypes. The single homozygous *PCBD1* p.(Glu97Lys) patient remains asymptomatic.

**Conclusion:** A higher proportion of BH4D (9.8 vs. 1%–2% worldwide), attributable to a heterogeneous mutational spectrum and wide clinical presentation, was noted in our Mexican HPA cohort, with the *PTS*-related HPA disorder being the most frequent. Sequencing-based assays could be a reliable approach for diagnosing BH4D in our population.

## 1 Introduction

An abnormally high blood level of the aromatic amino acid phenylalanine (Phe) is called hyperphenylalaninemia (HPA) and is mainly caused by deficiency of the enzyme phenylalanine hydroxylase (PAH, MIM*612349). To a lesser extent, HPA can be caused by genetically heterogeneous defects in the metabolism of the PAH cofactor tetrahydrobiopterin (BH4), accounting for 1%–2% of all HPA cases (Blau et al., 2010; Blau et al., 2018; Himmelreich et al., 2021), or deficiencies in the PAH chaperone DNAJ/HSP40 homolog, subfamily C, member 12 (MIM#617384), encoded by the *DNAJC12* gene (MIM*606060) (Blau et al., 2018; van Spronsen et al., 2021). In high-income countries, patients with HPA are mainly detected through newborn screening (NBS) programs. After a positive NBS test for HPA, the infant is routinely tested to differentiate between PAH and BH4 disorders (Opladen et al., 2012). This distinction is achieved by measuring the serum and urinary levels of neopterin, biopterin, and primapterin (pterin profile) and by evaluating dihydropteridine reductase (DHPR) activity (Opladen et al., 2011). However, the current wide availability of molecular testing has simplified the differential diagnosis of HPA, including BH4D (Carducci et al., 2020) (Table 1). Sepiapterin reductase (SR) deficiency and autosomal dominant *GCH1*-related disorder do not present elevated Phe, precluding their identification by NBS; hence, they can only be suspected by the clinical pictures in symptomatic patients (Blau, 2001).

**TABLE 1:** Genes and enzymes involved in BH4 metabolism disorders.

BH4 metabolism	Enzyme	MIM phenotype (#)	Gene	Number of exons	Mode of inheritance
Biosynthesis	GTP cyclohydrolase 1 (GTPCH)	233910	*GCH1*	6	AR
		128230	*GCH1*	6	AD
	6-pyruvoyltetra-hydropterin synthase (PTPS)	261640	*PTS*	6	AR
	Sepiapterin reductase (SR)	612716	*SPR*	3	AR
Regeneration	Pterin-4-alpha-carbinolamine dehydratase 1 (PCD)	264070	*PCBD1*	4	AR
	Dihydropteridine reductase (DHPR)	261630	*QDPR*	7	AR

GTP, guanidine triphosphate; AR, autosomal recessive; AD, autosomal dominant.

The specific HPA forms related to BH4D should be established as soon as possible, as the medical management is quite specific for each underlying enzymatic defect. Treatment includes the prescription of drugs such as L-DOPA, 5-hydroxytryptophan, and BH4 (sapropterin), among others (Opladen et al., 2020). In low- or middle-income countries, where HPA NBS programs are still under development, routine biochemical and/or molecular differential diagnosis between PAH and BH4D is not always available (Fernández-Lainez et al., 2018). BH4D can lead to uncommon, highly heterogeneous, and progressive neurological phenotypes attributable to neurotransmitter dysfunction, which are characteristically unresponsive to low-Phe nutritional treatment (Blau, 2001; Himmelreich et al., 2021).

Since 1996, Blau et al. (1996) have maintained the international BIOPKU database (BIOPKUdb, http://www.biopku.org), which is a repository of information devoted to the worldwide study of HPA. BIOPKUdb currently includes information on 1,161 patients with BH4D from various world regions (BIODEFdb, http://www.biopku.org/home/biodef.asp, accessed on 29th March 2022). Despite the large amount of information available in BIODEFdb, the molecular spectrum of BH4D in Mexican and Hispanic patients remains underrepresented.

Thus, the aim of this study is to report the genotypic spectrum underlying BH4D among all HPA patients seen at a single metabolic reference center in Mexico, to describe their frequency, to explore possible aspects of genotype-phenotype correlations and to perform *in silico* protein modeling of the deleterious effects expected for missense variants.

## 2 Materials and methods

### 2.1 Subjects

The patients studied herein were selected from a total of 142 unrelated Mexican individuals who were confirmed to have HPA (Phe blood levels >120 μmol/L) at our laboratory as previously described (Vela-Amieva et al., 2021). In 124 of these patients, the identification of biallelic *PAH* genotypes confirmed the underlying genetic etiology of their HPA deficiency; four patients showed a monoallelic *PAH* genotype, while in the remaining 14 patients, no *PAH* pathogenic variants were found (Vela-Amieva et al., 2021). These 14 patients were included in the present study, and their medical follow-up was available for periods ranging from 2 to 31 years. DHPR activity and blood and urine pterin profiles were available only in the four PTPS-deficient patients previously published (Fernández-Lainez et al., 2018). Herein, we provide updated medical follow-up. All patients were referred due to either an abnormal HPA NBS result or clinical data suggestive of metabolic disease. Clinical data, including type of HPA detection (NBS or high-risk metabolic screening), phenotype description, treatment used, outcome, and demographics, were recorded. To describe the phenotypic characteristics, the web-based application Phenomizer was used (https://hpo.jax.org/app/tools/phenomizer, accessed on 8th April 2022; Köhler et al., 2009). In patients under 6 years old, neurodevelopmental delay was assessed according to the Denver developmental screening test (Frankenburg et al., 1992). For older patients with intellectual disability, the Patel classification was used (Patel et al., 2018). The patients were categorized according to clinical severity as follows: asymptomatic (−), alive with moderate intellectual and motor disability (+), alive with profound intellectual and motor disability (++), and death with profound intellectual and motor disability (+++). This study was approved by the research, ethics, and biosecurity institutional committees (Registry 2020/014). Written informed consent was obtained from the legal guardians of the patients.

### 2.2 Molecular analysis by Sanger sequencing

Genomic DNA samples from the 14 HPA patients with a normal *PAH* genotype were obtained from dried blood spots by the saline precipitation method (Puregene kit; Gentra Systems, Minneapolis, MN, United States). These samples were further subjected to automated Sanger sequencing for four genes in the following order: *PTS* (NM_000317.3), *QDPR* (NM_000320.3), *PCBD1* (NM_000281.4), and *GCH1* (NM_000161.3). Parental samples from patients 5, 6, and 14 were directly genotyped to corroborate the *trans* configuration of the alleles. Polymerase chain reaction (PCR) amplification and direct automated Sanger sequencing were applied to cover the entire coding region and exon–intron borders (PCR oligonucleotides and sequencing conditions are available upon request). Identified variants were classified according to the scoring proposed by the American College of Medical Genetics and Genomics and the Association for Molecular Pathology (ACMG/AMP) (Richards et al., 2015). Computational evidence categories for predicting the deleterious effect of identified missense variants were obtained from Combined Annotation-Dependent Depletion (CADD) scoring (Rentzsch et al., 2019).

### 2.3 Comparison of frequencies of the pathogenic variants causing BH4D

The obtained data were compared with the information contained in BIODEFdb and other published literature indexed in MEDLINE/PubMed using the search terms BH4 deficiency, tetrahydrobiopterin, PTPS deficiency, DHPR deficiency and PCD deficiency.

### 2.4 *In silico* protein modeling

To predict the effects of the identified missense variants on the tertiary structures of the proteins, we performed *in silico* protein modeling. To that end, available crystallographic structures of human PTPS (PDB code 3I2B, https://www.rcsb.org) and DHPR (PDB code 1HDR) (Su et al., 1993) and rat PCD (PDB code 1DCP) were used. PyMOL Molecular Graphics System version 2.3.5 (Edu, Schrödinger, LLC, DeLano, 2002) was used to identify the locations of amino acid residues within the protein structures and to perform mutagenesis *in silico* to predict the potential consequences of single amino acid substitutions. To determine the grade of exposure of each mutant amino acid residue to the protein surface, the percentage of solvent-accessible surface area was calculated with the GETAREA tool (Fraczkiewicz and Braun, 1998, http://curie.utmb.edu/getarea.html).

## 3 Results

### 3.1 Subjects

All 14 included HPA patients with a normal *PAH* genotype had biallelic and clinically relevant genotypes highly indicative of underlying BH4D. Most of the patients were found to have a deficiency in PTPS (7/14, 50%), followed by DHPR (6/14, 42.9%) and PCD (1/14 7.1%). These results avoided the need for Sanger sequencing of the *GCH1* gene. A comparison of these frequencies with those reported in BIODEFdb is shown in Table 2. The mean Phe level at diagnosis of our BH4-deficient patients was 828 μmol/L (min 260 μmol/L, max 1,844 μmol/L) for PTPS-deficient patients, 645 μmol/L (min 470 μmol/L, max 799 μmol/L) for DHPR-deficient patients and 1,678 μmol/L for the PCD-deficient patient. The patients were from eleven different states of Mexico, with no specific region of the country predominating. Consanguinity was documented in only 3/14 patients (21.4%), and only patient 10 came from an endogamic region.

**TABLE 2:** BH4 defect variants found in the studied Mexican population.

BH4 enzymatic defect (gene)	Present study patients (%)	BIODEFdb				
		General	Caucasian	Spanish	Brazilian	Mexican/Other Hispanic
Number of patients	14	1,161	133	3	3	3/1
PTPS (*PTS*)	7 (50)	735 (63.3)	86*	2	1	1/1
DHPR (*QDPR*)	6 (42.90)	303 (26.09)	26	0	2	0/0
PCD (*PCBD1*)	1 (7.10)	30 (2.60)	12	1	0	2/0

*Considering all BH4D.

#### 3.1.1 Pharmacological treatment of patients

All the patients whose diagnostic Phe values were higher than 360 μmol/L were initially misdiagnosed as PAH deficiency and treated with a low Phe diet. Additionally, pharmacological treatment was initiated at a late stage in patients who received it. When BH4D was confirmed, PTPS-deficient patients 1, 2, 4, and 7 started pharmacological treatment according to the recommendations of Brennenstuhl et al., 2019, with L-Dopa at starting doses of 0.5–1 mg/kg/day to a maximum of 10 mg/kg/day combined with carbidopa at a 4:1 proportion. 5-Hydroxy-tryptophan was prescribed in a range from 1–8 mg/kg/day. Sapropterin dihydrochloride was indicated from 1 to 10 mg/kg/day according to the response of blood Phe levels. Due to the unavailability of pharmacological treatment, patient 3 received only nutritional therapy to control blood Phe levels. As the blood Phe levels of patients 5 and 6 were never higher than 360 μmol/L and they have remained asymptomatic, pharmacological therapy has not been started to date. DHPR-deficient patients received a similar treatment to PTPS patients, but instead of sapropterin dihydrochloride, they received nutritional treatment to control blood Phe levels, and 10–20 mg/day folinic acid was indicated. The PCD-deficient patient has not received any pharmacological treatment since his blood Phe levels have never been higher than 360 μmol/L after instauration of a low-Phe diet and he remains asymptomatic to date.

### 3.2 Genotypic spectra of the *PTS*, *QDPR*, and *PCBD1* genes

A total of 14 clinically relevant variants were found (Table 3). Missense variants were the most frequent at 46.42% (13/28 alleles). Four previously undescribed variants were identified, all in *QDPR*: c.714dup or p.(Leu239Thrfs*44), c.106-1G>T or p.(?), c.214G>T or p.(Gly72*), and c.187_189dup or p.(Gln63dup). The most common variants observed in *PTS* were c.331G>T or p.(Ala111Ser) and c.393del or p.(Val132Tyrfs*19), with four alleles each.

**TABLE 3:** Clinically relevant variants identified in Mexican HPA patients with suspected BH4 deficiency.

Gene	Variant	Protein change	Alleles (n)	Location	Coding effect	ACMG/AMP variant classification	CADD score	GnomAD allele frequencies in healthy subjects	First reported in BH4D patients
*PTS* (14 alleles)	c.200C>T	p.(Thr67Met)	1	Ex 4	Missense	Pathogenic	31	0.007	Oppliger et al. (1997)
	c.338A>G	p.(Tyr113Cys)	2	Ex 6	Missense	Pathogenic	20.7	0.0028	Meli et al. (1999)
	c.242A>C	p.(Glu81Ala)	1	Ex 4	Missense	Likely Pathogenic	25.5	0.0007	Present study
	c.331G>T	p.(Ala111Ser)	4	Ex 6	Missense	Likely Pathogenic	23.5	Not reported	Fernandez-Laninez et al. (2018)
	c.73C>T	p.(Arg25*)	2	Ex 1	Nonsense	Pathogenic	38	0.001	Chiu (2012)
	c.393del	p.(Val132Tyrfs*19)	4	Ex 6	Frameshift	Pathogenic	26.1	0.0015	Himmelreich et al. (2021)
*QDPR* (12 alleles)	**c.106-1G>T**	**p.(?)**	2	In 1	Splice site	Pathogenic	35	Not reported	Present study
	**c.187_189dup**	**p.(Gln63dup)**	1	Ex 2	In-frame	Likely Pathogenic	Not obtained	Not reported	Present study
	**c.214G>T**	p.(Gly72*)	2	Ex 3	Nonsense	Pathogenic	23.7	Not reported	Present study
	c.482G>T	p.(Cys161Phe)	1	Ex 5	Missense	Likely Pathogenic	25.6	0.0003	Present study
	c.515C>T	p.(Pro172Leu)	2	Ex 5	Missense	Likely Pathogenic	25.6	0.002	Lu et al. (2014)
	**c.714dup**	p.(Leu239Thrfs*44)	2	Ex 7	Frameshift	Pathogenic	Not obtained	Not reported	Present study
	c.661C>T	p.(Arg221*)	2	Ex 7	Nonsense	Pathogenic	41	0.0011	Smooker et al. (1993)
*PCBD1* (2 alleles)	c.289G>A	p.(Glu97Lys)	2	Ex 4	Missense	Likely Pathogenic	24.1	0.0014	Thöny, Neuheiser et al. (1998)

New variants are highlighted in bold. ACMG/AMP, American college of medical genetics and genomics/association of molecular pathology (Richards et al., 2015); CADD, combined annotation dependent depletion scoring (Rentzsch et al., 2019); GenomAD, genome aggregation database.

### 3.3 Genotype/phenotype descriptions

The clinical characteristics of each patient and their *PTS*, *QDPR*, or *PCBD1* genotype are presented in Table 4.

**TABLE 4:** Genotype/phenotype description of Mexican patients bearing clinically relevant biallelic *PTS*, *QDPR*, and *PCBD1* genotypes.

		*PTS* genotype							*QDPR* genotype						*PCBD1* genotype
Patient		1	2^✦^	3^✦^	4^✦^	5	6	7^✦^	8	9	10	11	12	13	14^a^
Sequence variation	Allele 1	c.200C>T	c.331G>T	c.331G>T	c.331G>T	c.338A>G	c.338A>G	c.393delA	**c.106-1G>T**	**c.187_189dup**	**c.214G>T**	c.515C>T	c.661C>T	**c.714dup**	c.289G>A
	Allele 2	c.242A>C	c.331G>T	c.73C>T	c.73C>T	c.393delA	c.393delA	c.393delA	**c.106-1G>T**	c.482G>T	**c.214G>T**	c.515C>T	c.661C>T	**c.714dup**	c.289G>A
Protein change	Protein change 1	p.(Thr67Met)	p.(Ala111Ser)	p.(Ala111Ser)	p.(Ala111Ser)	p.(Tyr113Cys)	p.(Tyr113Cys)	p.(Val132Tyrfs*19)	p.(?)	p.(Gln63dup)	p.(Gly72*)	p.(Pro172Leu)	p.(Arg221*)	p.(Leu239Thrfs*44)	p.(Glu97Lys)
	Protein change 2	p.(Glu81Ala)	p.(Ala111Ser)	p.(Arg25*)	p.(Arg25*)	p.(Val132Tyrfs*19)	p.(Val132Tyrfs*19)	p.(Val132Tyrfs*19)	p.(?)	p.(Cys161Phe)	p.(Gly72*)	p.(Pro172Leu)	p.(Arg221*)	p.(Leu239Thrfs*44)	p.(Glu97Lys)
Variant classification	Allele 1	Pathogenic	Likely pathogenic	Likely pathogenic	Likely pathogenic	Pathogenic	Pathogenic	Pathogenic	Pathogenic	Likely pathogenic	Pathogenic	Likely pathogenic	Pathogenic	Pathogenic	Likely pathogenic
	Allele 2	Likely pathogenic	Likely pathogenic	Pathogenic	Pathogenic	Pathogenic	Pathogenic	Pathogenic	Pathogenic	Likely pathogenic	Pathogenic	Likely pathogenic	Pathogenic	Pathogenic	Likely pathogenic
Phenotype/clinical description															
Family history	Consanguinity				+									+	+
	Endogamy										+				
	Previous death siblings									+					
Prenatal	Oligohydramnios (HP: 0001562)			+											
Neonatal	Preterm birth							+							
	Small for gestational age			+	+			+							
Neurological	Seizures (HP:0001250)	+	+		+			+	+	+	+	+	+	+	
	Dystonia (HP:0001332)	+	+	+	+			+	+	+	+	+	+	+	
	Irritability (HP: 0000737)	+		+	+										
	Drowsiness (HP: 0002329)	+		+	+										
	Severe muscular hypotonia (HP: 0006829)								+	+	+	+	+	+	
	Neurodevelopmental (HP: 0012758) and motor delay (HP:0001270)	+	+	+	+			+	+	+	+	+	+	+	
Extrapyramidal signs HP:0002071	Excessive salivation (HP: 0003781)	+	+	+	+				+	+	+	+	+	+	
	Abnormality of eye movement (HP: 0000496)		+					+	+	+	+	+	+	+	
	Intermittent hyperventilation (HP: 0004879)		+					+	+	+	+	+	+	+	
	Hyperreflexia (HP: 0001347)		+					+	+	+	+	+	+	+	
Dysmorphias	Microcephaly (HP: 000252)	+			+			+	+						
	Dolichocephaly (HP: 0000268)							+							
	Dysmorphological syndrome								^b^+						
Others	Failure to thrive (HP: 0001508)		+					+							
	Hair hypopigmentation (HP: 0005599)		+		+			+			+			+	
	Hypopigmentation of the skin (HP: 0001010)		+		+			+			+			+	
	Necrotizing enterocolitis							+							
	Unexplained fevers (HP: 0001955)	+		+	+										
	Dysphagia (HP: 0002015)		+					+	+	+	+	+	+	+	
	Kidney calcifications								+						
HPA diagnosis		HRMS	NBS	NBS	HRMS	NBS	NBS	HRMS	NBS	NBS	HRMS	HRMS	NBS	NBS	NBS
Biochemical data	Initial blood Phe (μM) (RV: <120)	1844	738	1027	NA	260	307	820	1771	711	742	546	470	799	1672
Clinical severity		++	+	+++	+++	−	−	++	++	++	++	++	+++	++	−
Outcome		Alive, 24 years old, with profound intellectual disability and motor impairment	Alive, 10 years old, with moderate intellectual disability and motor Impairment	Death at 3 years old with profound neurodevelopmental impairment	Death at 14 years old with profound intellectual disability and motor impairment	Alive, 6 years old, asymptomatic	Alive, 10 years old, asymptomatic	Alive, 12 years old, with moderate intellectual disability and motor impairment	Alive, 2 years old, with profound neurodevelopmental impairment	Alive, 3 years old, with profound neurodevelopmental impairment	Alive, 19 years old, with profound intellectual disability and motor impairment	Alive, 31 years old, with profound intellectual disability and motor impairment	Sudden death at 2 years old and profound neurodevelopmental impairment	Alive, 4 years old, with profound neurodevelopmental impairment	Alive, 3 years old, asymptomatic

HP, phenomizer code; HPA, hyperphenylalaninemia; HRMS, high-risk metabolicscreening; NA, not available; NBS, newborn screening; RV: Reference value. ✦ previously reported Fernandez-Laninez et al., 2018. New variants are highlighted in bold.

^a^ Blood glucose (mg/dl) (RV: 57–115): 75. Blood magnesium (mg/dl) (RV: 1.7–2.3): 2.1. Blood total cholesterol (mg/dl) (RV: 114–203): 249. Blood LDL, cholesterol (mg/dl) (RV: 38–140): 154.4.

^b^ Bilateral radial alterations, renal agenesis without specific diagnosis. Normal karyotype in 15 metaphases with 450–500 bands of resolution.

#### 3.3.1 *PTS* genotypes

Among the five different *PTS* genotypes identified in seven affected individuals, five were compound heterozygous (patients 1, 3, 4, 5, and 6), and two of them were homozygous (patients 2 and 7, Table 4). Five of seven patients (71.4%) developed neurological impairment, while in the two remaining ones (patients 5 and 6) with the c.338A>G or p.(Tyr113Cys) and c.393delA or p.(Val132Tyrfs*19) variants remain asymptomatics to date. To improve delineation of the pathogenicity of the p.(Tyr113Cys) variant and to confirm their compound heterozygous *PTS* genotypes, the carrier status of their parents for each variant was corroborated. The mortality rate of PTPS-deficient patients was 28.6% (2/7). Remarkably, both patients had the same p.[Arg25*]; [Ala111Ser] predicted genotype.

#### 3.3.2 *QDPR* genotypes

Five of the six different genotypes of DHPR-deficient patients (Table 4) were homozygous; nevertheless, consanguinity or endogamy was documented only in patients 10 and 13. These two families did not share a common geographical origin. All the studied patients developed severe neurological impairment, with a mortality rate of 16.7% (1/6). The deceased patient was homozygous for the p.(Arg221*) variant.

#### 3.3.3 *PCBD1* genotype

Only one pathogenic homozygous *PCBD1* genotype was identified in a patient whose parents were consanguineous (Table 4, patient 14). The obligate heterozygous genotype was confirmed only in his mother. This patient was detected by NBS with a Phe value of 1,672 μmol/L, which was normalized after initiation of a low-Phe diet. His dietary tolerance for this amino acid was later improved. Currently, he is 3 years old, on a normal diet, with a weight Z score of −0.97, a height Z score of −1.99, and normal neurodevelopmental outcomes. At this moment, the patient has not shown evidence of early-onset nonautoimmune (MODY)-type diabetes or hypomagnesemia.

### 3.4 *In silico* protein modeling

#### 3.4.1 Modeling of PTPS variants

**FIGURE 1:**
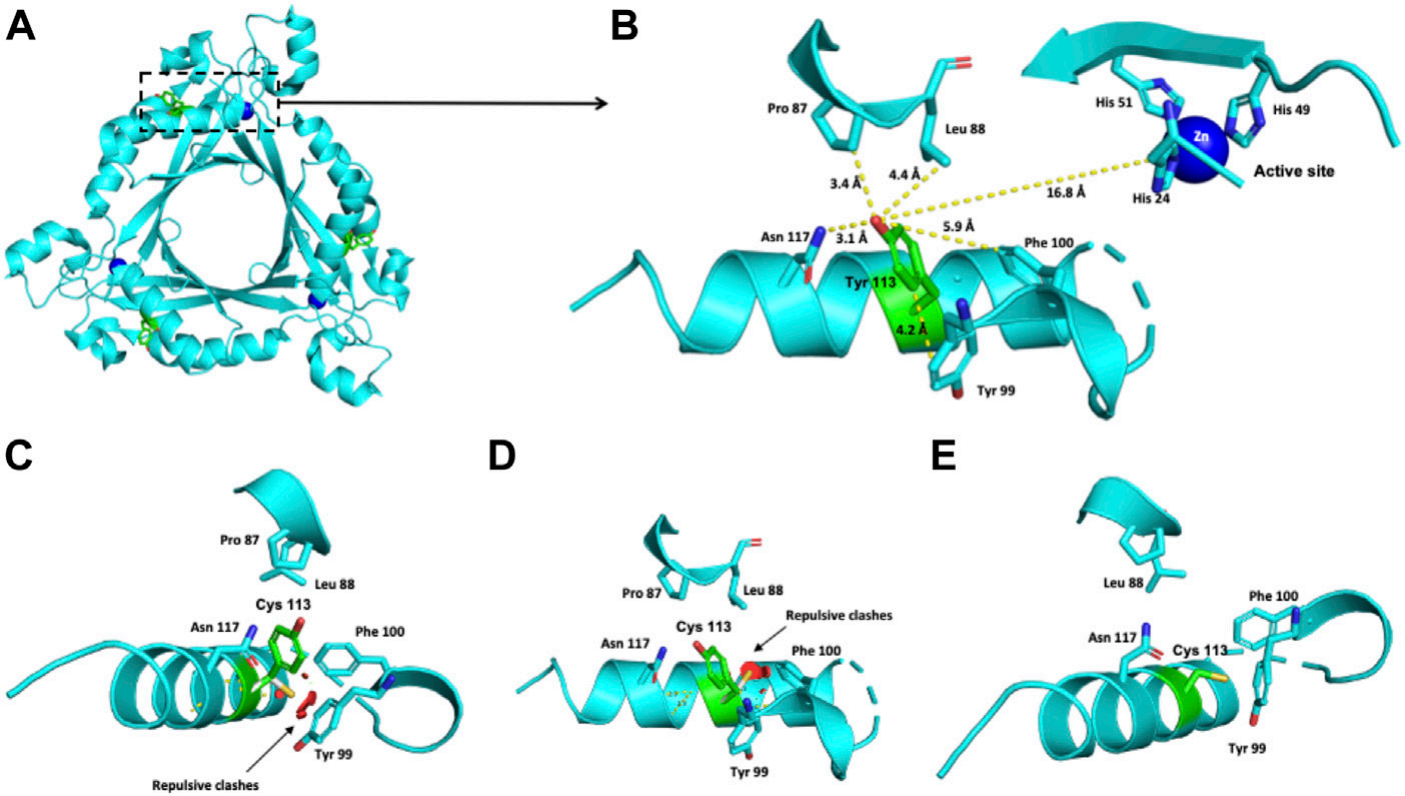
Modeling of the human PTPS structure with p.(Tyr113Cys) change. **(A)** Crystallographic structure of a trimer of PTPS and the location of the Tyr 113 residue on each monomer. **(B)** Location of the Tyr 113 residue in one monomer. **(C–E)** Potential pathogenic effects for the substitution of Tyr by Cys.

**FIGURE 2:**
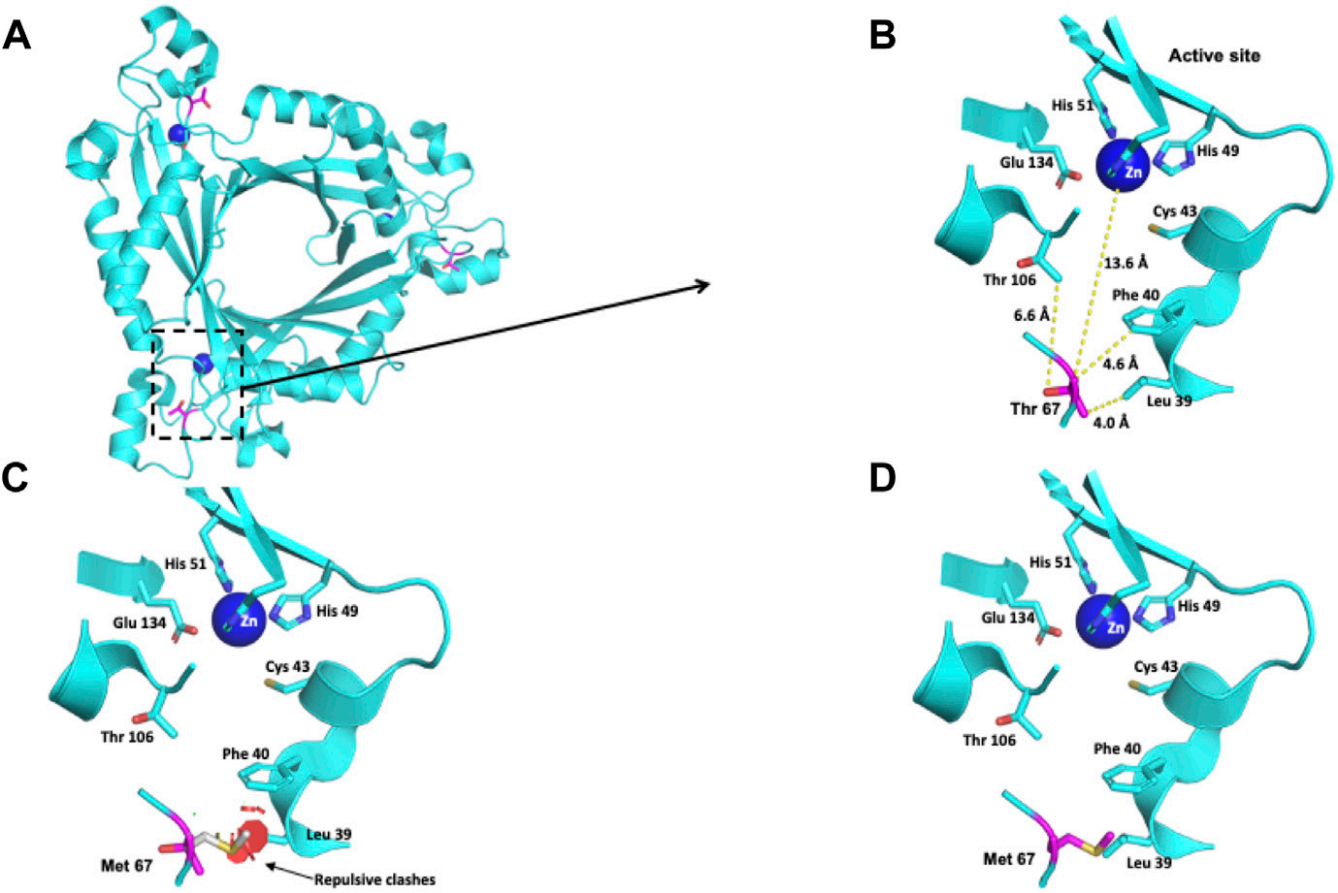
Modeling of the human PTPS structure with p.(Thr67Met) change. **(A)** Crystallographic structure of a trimer of PTPS and location of the Thr 67 residues on each monomer. **(B)** Location of the Thr 67 residue in one monomer. **(C–D)** Potential pathogenic effects for the substitution of Thr by Met.

**FIGURE 3:**
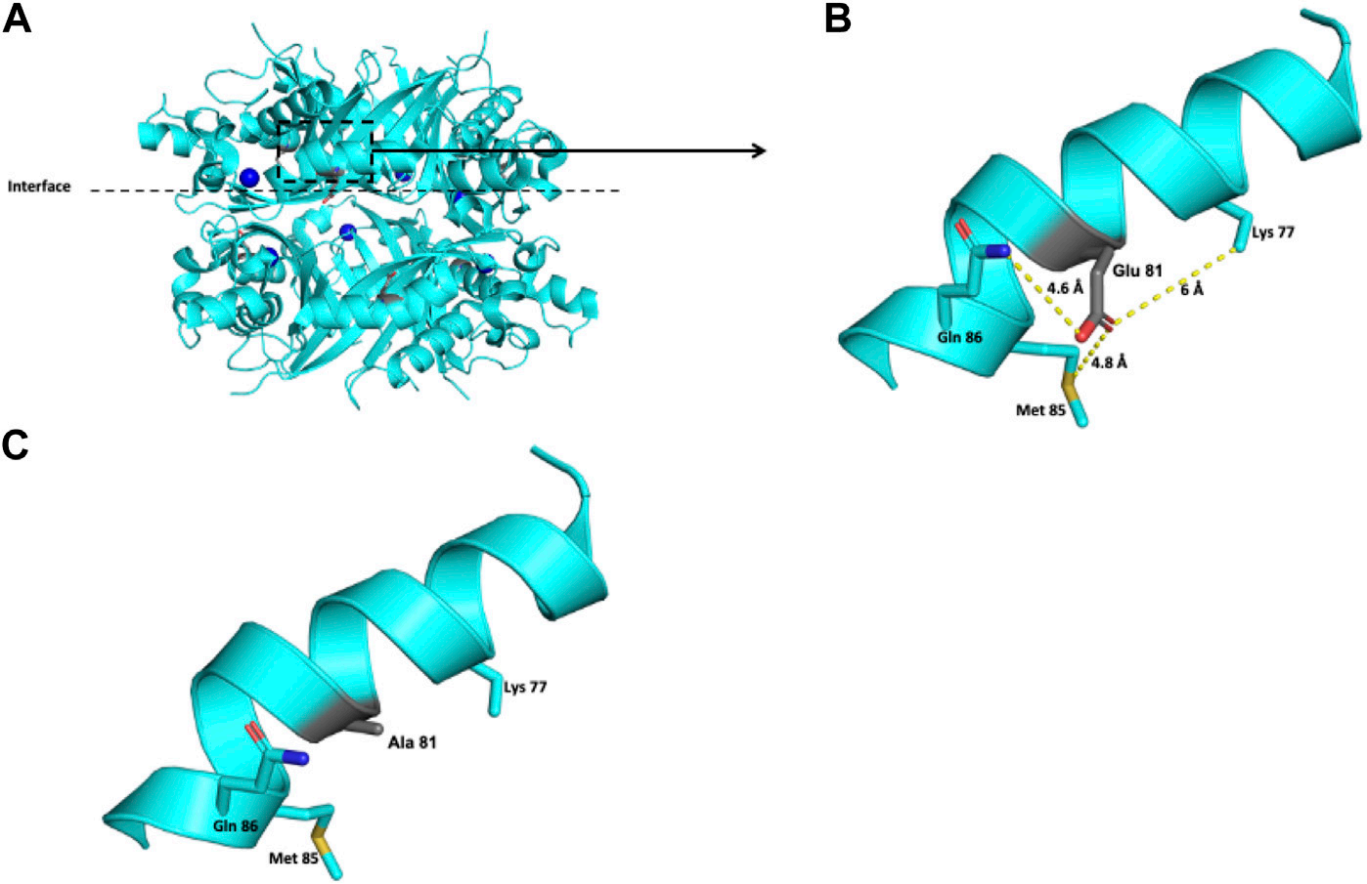
Modeling of the human PTPS structure with p.(Glu81Ala) change. **(A)** Crystallographic structure of a trimer of PTPS and location of the Glu 81 residues on each monomer. **(B)** Location of the Glu 81 residue in one monomer. **(C)** Potential pathogenic effect for the substitution of Glu by Ala.

Three missense variants, p.(Thr67Met), p.(Glu81Ala), and p.(Tyr113Cys), were modeled (Figures 1–3). The most common likely pathogenic variant, p.(Ala111Ser), was previously modeled by our group (Fernández-Lainez et al., 2018).

The Thr residue at position 67 is 13.6 Å away from the active site (Figures 2A,B). This polar uncharged residue interacts with the Thr 106, Leu 39 and Phe 40 residues (Figure 2B). When Thr 67 is changed to a nonpolar Met residue, repulsive interactions between this residue and the nonpolar residue Leu 39 occur (Figures 2C,D).

The Glu 81 residue is located at the interface between the two trimers that conform to the functional unit of the PTPS protein (Figure 3A). This residue has a solvent-accessible surface area of 54.6%. In a 6-Å radius, this negatively charged residue is surrounded by the polar Gln 86, Glu 81, and Lys 77 residues and by the nonpolar Met 85 residue (Figure 3B). The exchange of this polar residue for the aliphatic alanine provokes the loss of the negative charge that Glu contributes to that environment (Figure 3C).

The crystallographic structure of a trimer of PTPS and the location of the Tyr 113 residue on each monomer are shown in Figure 1A. The Tyr 113 residue of the PTPS protein is located 16.8 Å from the active site (Figure 1B). Within 3–6 Å, it is surrounded by Pro 87, Leu 88, Asn 117, Tyr 99 and Phe 100 residues (Figure 1B). This environment is hydrophobic and nonpolar. The exchange of this Tyr residue for the polar cysteine residue provokes repulsive interactions with the nonpolar Tyr 99 and Phe 100 residues (Figures 1C–E).

#### 3.4.2 Modeling of DHPR variants

**FIGURE 4:**
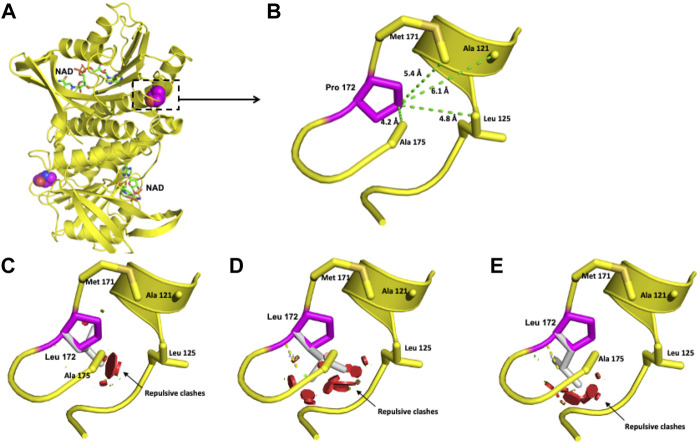
Modeling of the human DHPR structure with p.(Pro172Leu) change. **(A)** Crystallographic structure of a dimer of DHPR and location of the Pro 172 residues on each monomer. **(B)** Location of the Pro 172 residue in one monomer. **(C–E)** Potential disturbing structural effects for the substitution of Pro 172 by Leu.

For the DHPR protein, two missense variants, p.(Cys161Phe) and p.(Pro172Leu), classified as likely pathogenic were modeled. The crystallographic structure of the functional unit of DHPR is shown in Figure 4A.

**FIGURE 5:**
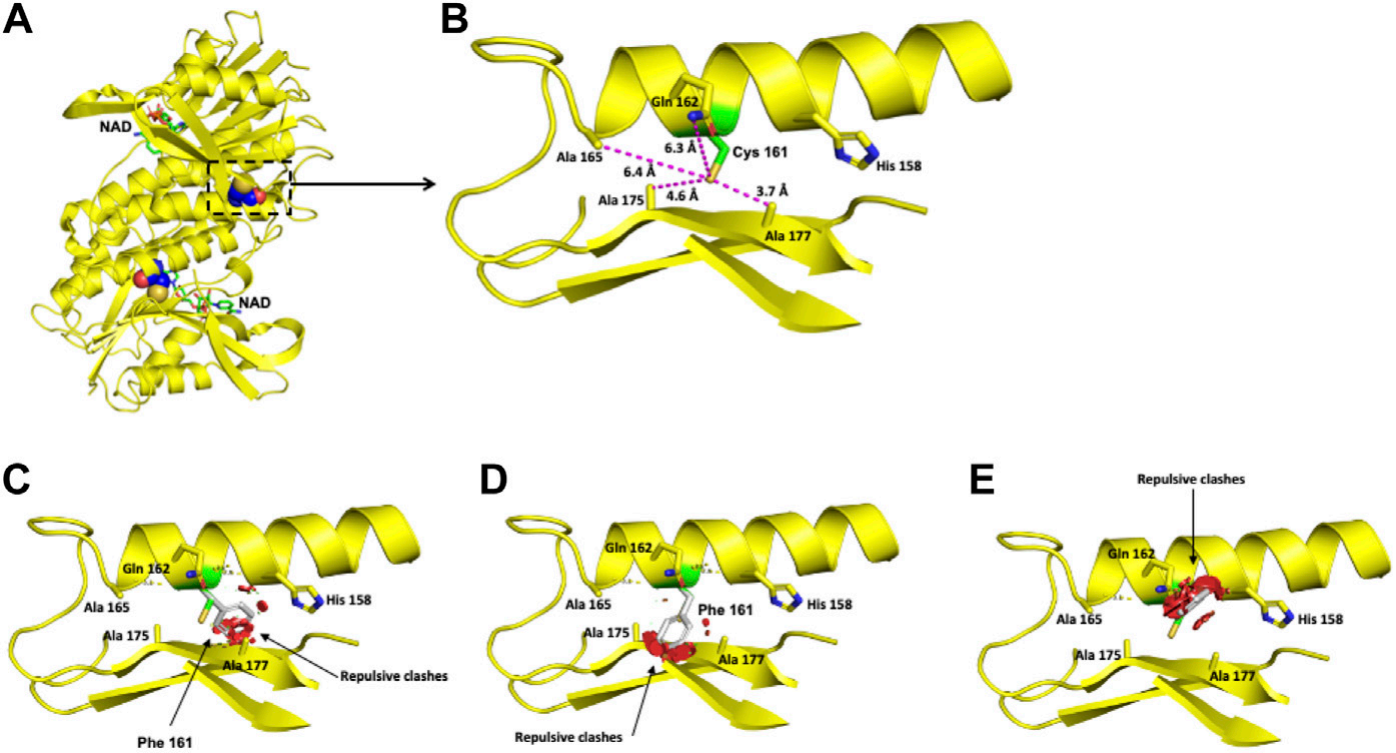
Modeling of the human DHPR structure with p.(Cys161Phe) change. **(A)** Crystallographic structure of a dimer of DHPR and location of the Cys 161 residues on each monomer. **(B)** Location of the Cys 161 residue in one monomer. **(C–E)** Potential pathogenic effects for the substitution of Cys by Phe.

The polar Cys 161 residue is surrounded in a 6-Å radius by the nonpolar Ala 165, Ala 175, and Ala 177 residues and the polar Gln 162 residue (Figures 5A,B). Exchanging the polar Cys residue for the hydrophobic nonpolar Phe provokes repulsive interactions with the surrounding amino acid residues (Figures 5C-E).

The Pro 172 residue is surrounded by Ala 175, Leu 125, Ala 121, and Met 171 in a 6-Å radius (Figure 4B). This residue has an 84.5% solvent-accessible surface area. The exchange of this Pro residue for an Ala residue provokes repulsive interactions in that zone. Although both residues are nonpolar, alanine extends its linear aliphatic chain, while the Pro ring does not extend (Figures 4C–E).

#### 3.4.3 Modeling of the p.(Glu97Lys) PCD variant

**FIGURE 6:**
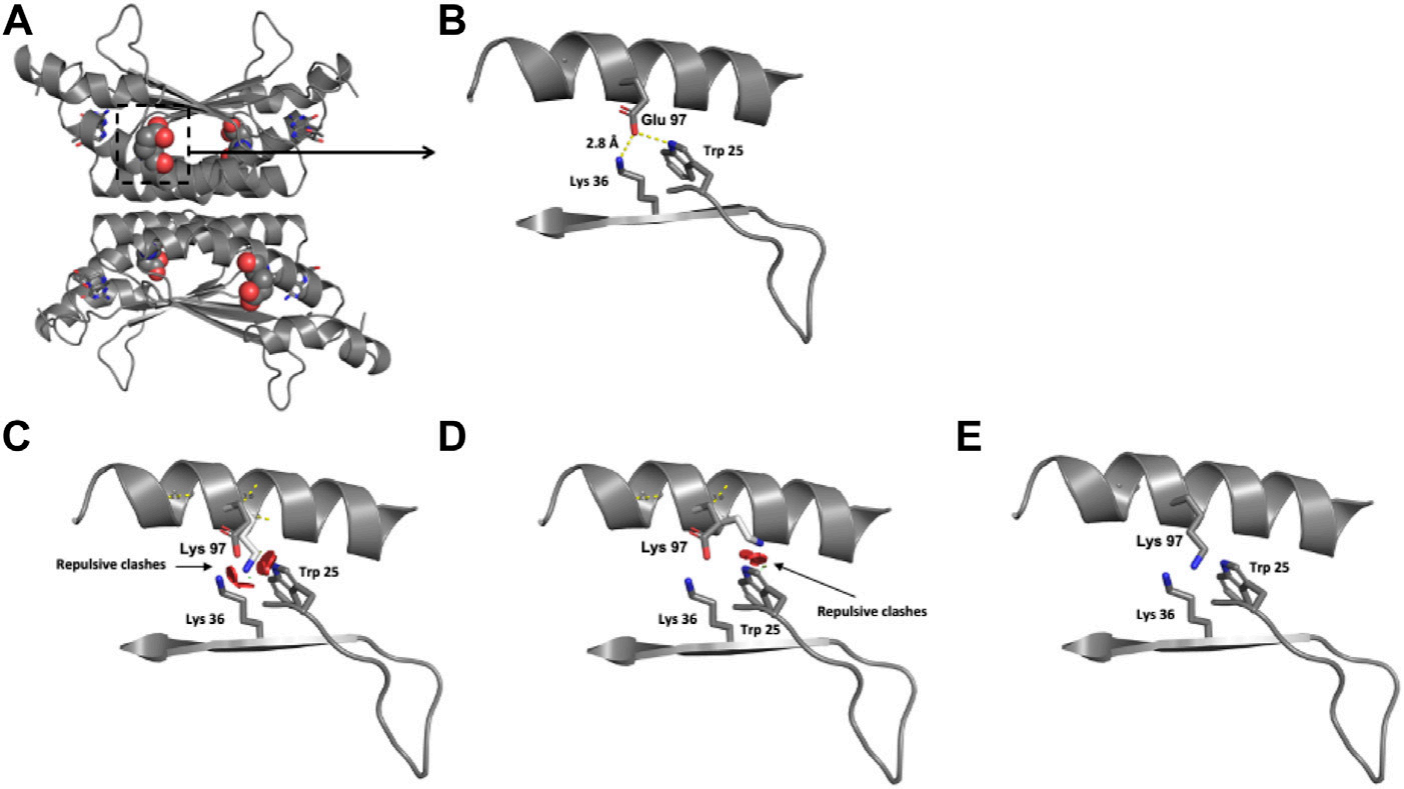
Modeling of human PCD structure with p.(Glu97Lys) change. **(A)** Crystallographic structure of a dimer of PCBD1 and location of the Glu 97 residues on each monomer. **(B)** Location of the Glu 97 residue in one monomer. **(C–E)** Potential pathogenic effects for the substitution of Glu by Lys.

The location of the Glu 97 residue in the crystallographic structure of the functional unit of PCD is shown in Figure 6A. Modeling of the single identified *PCBD1* missense variant reveals that the negatively charged Glu 97 residue is surrounded by the positively charged Lys 36 residue and the hydrophobic Trp 25 residue, both within 3 Å (Figure 6B). The exchange of this negatively charged residue for the positively charged Lys provokes repulsive interactions with the neighboring Lys 36 and Trp 25 residues (Figures 6C-E).

## 4 Discussion

This study describes for the first time a prevalence of 9.8% for BH4D in a cohort of Mexican patients with HPA. Despite the low number of patients analyzed, we documented a heterogeneous molecular landscape of BH4D, revealing four novel variants, all in *QDPR*. Our results demonstrate that pathogenic variants responsible for BH4D contribute to an important proportion of HPA patients. This value of 9.8% for BH4D appears to be higher than that described worldwide (1–2%, Blau et al., 2018; Himmelreich et al., 2021). However, a higher prevalence of BH4D has also been reported in populations in other areas, such as Jordan (33%, Carducci et al., 2020), the southern region of China (15.1%, Yuan et al., 2021), and Iran (12.3%, Khatami et al., 2016). We speculate that the high frequency of BH4D in this sample of Mexican HPA patients could be related to bias, since our institution serves patients with abnormal NBS to perform confirmatory diagnosis, management, and medical follow-up. We are also a tertiary medical referral center for symptomatic patients suspected to have inborn metabolic diseases. In other populations with a high prevalence of BH4D, a high incidence of consanguinity or endogamy (57%) has been found as a possible explanation (Carducci et al., 2020); remarkably, in the present study, there was a lower proportion of families with consanguinity or endogamy (4/14 families, 28.5%, Table 4). Moreover, the hypothesis of a founder effect for the c.331G>T or p.(Ala111Ser) variant in the southeastern region of Mexico (Fernández-Lainez et al., 2018), could not be demonstrated here, but further studies are warranted in a larger number of patients.

The Sanger methodology allowed us to determine clinically relevant biallelic *PTS*, *QDPR*, and *PCBD1* genotypes in all 14 patients analyzed. Missense variants accounted for the most frequent changes observed (13/28, 46.42%) (Table 3). This finding agrees with previously published data, where *PTS*, *QDPR*, and *PCBD1* missense variants accounted for 50.5% of the 800 variants responsible for BH4D worldwide (Himmelreich et al., 2021). By assuming that clinically relevant *PTS*, *QDPR*, and *PCBD1* genotypes were identified in all 14 patients, this finding suggests that *GCH1*, *SR*, and *DNAJC12* defects would be rare conditions underlying HPA in our population.

The present study shows the complexity of establishing genotype-phenotype correlations of BH4D and the severe neurological impairment (11/14 patients, 78.6%), as well as a high mortality rate (21.4%) as documented in other populations (Blau et al., 1996; Opladen et al., 2012), especially when prompt diagnosis and treatments are not achieved, highlighting the importance of NBS and differential diagnosis of HPA. Compared to Sanger sequencing, high-throughput sequencing platforms applied to NBS are a promising tool that is being introduced in high-income countries, despite all the challenges it represents (Watson et al., 2022). However, its availability seems barely feasible in low- and middle-income countries in the near future; thus, Sanger sequencing would remain as an alternative for BH4D genotyping.

### 4.1 *PTS* variants and genotypes

The predominance of PTPS defects responsible for BH4D (50%) is comparable to that reported in BIODEFdb, where its prevalence is 63.3% (BIODEFdb). *PTS* exons 4 and 6 were the most frequently affected, with two variants each. Exon 6 has also been reported to have a high proportion of pathogenic variants (Himmelreich et al., 2021). Five different *PTS* genotypes were observed (Table 4). To date, identical genotypes have not been reported in BIODEFdb or the literature.

The patient with the c.[200C > T];[242A > C] or p.[Thr67Met];[Glu81Ala] *PTS* genotype (Patient 1, Table 4) was severely affected. The p.(Thr67Met) variant was previously reported in a homozygous state in an Italian patient who also had a severe form of PTPS deficiency attributable to residual enzymatic activity lower than 1% (Oppliger et al., 1997). Our protein modeling suggests an effect on the catalytic site at long distances (Figure 2). The p.(Glu81Ala) variant has a low allele frequency (0.0007) in gnomAD; however, to the best of our knowledge, it has not been reported as an allele responsible for PTPS deficiency. In this variant, the substitution of polar, negatively charged glutamic acid 81 for nonpolar, neutral Ala decreases the negative charge in a 6-Å radius environment that surrounds the Glu 81 residue. Since this residue is in a superficial location, the change could interfere with the interaction necessary between the two trimers to conform to the PTPS functional unit (Figure 3). Altogether, these clinical and structural findings support the pathogenicity of this genotype.

The most severe phenotypes observed in the present study were those of patients bearing the compound heterozygous *PTS* genotype c.[73C > T];[331G > T] or p.[Arg25*];[Ala111Ser], which were identified in two nonrelated patients (patients 3 and 4, Table 4) with fatal outcomes. Therefore, the presence of the predicted null p.(Arg25*) allele would be considered responsible for the fatal phenotype observed.

A less severe clinical picture of PTPS deficiency was observed in nonrelated patients 5 and 6 (Table 4). They had the same *PTS* genotype, c.[338A > G];[393del] or p.[Tyr113Cys]; [Val132Tyrfs*19]. The carrier status of their parents was determined, which corroborates the *trans* configuration of both alleles in each patient.

We also performed molecular modeling of the c.338A>G or p.(Tyr113Cys) variant. Despite the repulsive clashes observed when substituting Cys for the Tyr 113 residue (Figures 1C-E), these patients showed no signs of disease and maintained normal neurological development. Similar observations were made by Meli et al. (1999) in a consanguineous Sicilian family with the homozygous p.(Tyr113Cys) variant; their three patients initially presented with HPA, which was eventually self-limited, and they also had normal neurodevelopment. Moreover, another patient bearing the p.[Pro87Leu];[Tyr113Cys] *PTS* genotype was reported to have a peripheral form. This patient was detected by the presence of HPA without any clinical signs (Preuße, 2001). Thus, the p.(Tyr113Cys) variant either in a homozygous or heterozygous state seems to lead to a benign phenotype. Further functional studies are warranted to further clarify the level of residual enzymatic activity of p.(Tyr113Cys) and how it can be correlated with a benign clinical outcome. Moreover, the low pathogenicity of this variant could attenuate the null effect expected for the second frameshift p.(Val132Tyrfs*19) allele in these patients. This can be supported by our findings in patient 7, homozygous for the c.393delA or p.(Val132Tyrfs*19) variant, which show that during 12 years of medical follow-up, he had moderate intellectual and motor disabilities, although he started pharmacological treatment when he was 5 months old. This highlights the difficulties in the establishment of the genotype-phenotype correlation considering variables such as age at the beginning of treatment.

### 4.2 *QDPR* variants and genotypes

DHPR defects accounted for 42.9% of the BH4D cases identified herein, which is higher than the 26.09% described in BIODEFdb (BIODEFdb). Of the seven *QDPR* variants found (Table 3), five were single base substitutions, which coincides with the findings reported by Himmelreich et al. (2021), where this nucleotide change predominated (112 base substitutions among the 141 *QDPR* pathogenic variants). Most of them were located in exon 5, while in the present study, the variants were distributed along exons 1, 2, 3, 5, and 7.

The clinical picture observed in the four patients bearing the four new *QDPR* alleles allows us to consider these variants responsible for severe phenotypes. Moreover, the protein modeling of the two likely pathogenic variants, c.515C > T or p.(Pro172Leu) and c.482G > T or p.(Cys161Phe), reveals potential damage to the structure of the protein (Figures 4, 5), which correlates with the observed severe phenotype. Five of the six *QDPR* genotypes were homozygous (Table 4). Although high numbers of homozygous *QDPR* pathogenic genotypes seem to be common among other populations with high consanguinity (Romstad et al., 2000; Foroozani et al., 2015), this feature or endogamy was found in only 33.3% (2/6 *QDPR* families) in this study. Moreover, none of the families share family names, and they come from different geographic regions of the country.

Regardless of the identified *QDPR* genotype, all these patients showed similarly severe phenotypes (Table 4). This clinical picture has been exhaustively described in the literature (Blau, 2001; Himmelreich et al., 2021). However, none of the *QDPR* genotypes presented here, except for the p.[Arg221*];[Arg221*] genotype (Dianzani et al., 1998; Khatami et al., 2016), have been previously reported. The c.515C > T or p.(Pro172Leu) variant identified in the homozygous state in patient 11 (Table 4) has been previously described only once in a Chinese patient who presented severe neurological symptoms and had a compound heterozygous p.[Pro172Leu];[Arg221*] genotype (Lu et al., 2014). Thus, this evidence supports the conclusion that c.515C > T or p.(Pro172Leu) could be considered a severe allele.

The notion of severity of the c.661C > T or p.(Arg221*) variant was supported by the clinical picture documented in homozygous patient 12 (Table 4), who suddenly died at 2 years old after presenting the characteristic clinical picture of DHPR deficiency (Blau, 2001). This variant has also been described in severely affected patients, where it was considered a null allele that led to an inactive or highly degraded enzyme (Smooker et al., 1993). Moreover, an identical genotype has been reported in at least three patients who also had a severe phenotype; however, their outcomes were not reported (Dianzani et al., 1993; Dianzani et al., 1998; Khatami et al., 2016).

As a possible explanation of the fatal outcomes of these severe phenotypes, it has been proposed that accumulation of dihydrobiopterin (BH_2_) leads to inhibition of aromatic amino acid hydroxylases and dysregulation of nitric oxide metabolism, which diminishes cerebral 5-methylenetetrahydrofolate levels (Opladen et al., 2012; Himmelreich et al., 2021).

### 4.3 *PCBD1* variant and genotype

The frequency of PCD deficiency in the present study was 7.1%, exceeding the 2.6% reported in BIODEFdb (BIODEFdb), which could suggest that the frequency of the different BH4 disorders varies among populations (Souza et al., 2018; Carducci et al., 2020; Bozaci et al., 2021; Gundorova et al., 2021; Ray et al., 2022). We only found one homozygous c.289G > A or p.(Glu97Lys) male patient. To date, an identical genotype has not been reported. The protein modeling of the variant predicts a potential charge disturbance at the site of the Glu 97 residue, which deserves further study. Due to the dual activity of the coded product of *PCBD1*, this BH4D has been related to early-onset nonautoimmune (MODY)-type diabetes and hypomagnesemia due to renal magnesium wasting (Simaite et al., 2014; Himmelreich et al., 2021). However, this variant has only been previously associated with MODY-type diabetes but not with hypermagnesuria (Ferrè et al., 2014). To date, our three-year-old patient remains asymptomatic with a close follow-up to detect these potential complications.

### 4.4 Limitations

One limitation of this study is the unavailability of the pterin profile for blood, urine, or cerebrospinal fluid in our patients. The lack of this information precludes the appropriate definition of the subtypes and phenotypic characteristics of each BH4D, such as the peripheral forms of PTPS deficiency. It is important to note that our inclusion criteria relied on the sole presence of HPA along with a normal *PAH* genotype, and BH4 defects such as SR and GTPCH deficiencies that do not appear in HPA could not be detected with this approach. Additionally, to determine the real birth prevalence and genotypic spectrum of BH4D in our population, it would be necessary to establish their detection through universal NBS-based programs, which could prevent any bias due to the selection of high-risk patients in a specialized medical unit.

## 5 Conclusion

BH4D was found in 9.8% of HPA cases in a sample of Mexican patients who attended a single Mexican reference center for inborn metabolic diseases. Our molecular approach led us to identify clinically relevant and biallelic *PTS*, *QDPR* and *PCBD1* genotypes in HPA patients bearing a normal *PAH* genotype. The identified mutational spectrum was heterogeneous, with PTPS deficiency being the most frequent (50% of these cases), followed by DHPR deficiency (42.9%), which was associated with the worst clinical course and outcome, including death at an early stage of life. Instead, in PTPS and PCD deficiencies, two and one asymptomatic patients were documented, respectively. It is essential to strengthen NBS programs in low- and middle-income countries to include the differential biochemical or molecular detection for BH4D, since late diagnosis could lead to a poor prognosis. Furthermore, the current massive sequencing platforms could facilitate prompt differential BH4D diagnosis.

## Data Availability

The original contributions presented in the study are included in the article/supplementary material, further inquiries can be directed to the corresponding author.
